# The importance of the size of the trunk inclination angle in the early detection of scoliosis in children

**DOI:** 10.1186/s12891-021-04965-4

**Published:** 2022-01-03

**Authors:** Marek Kluszczyński, Anna Pilis, Dariusz Czaprowski

**Affiliations:** 1 Department of Health Sciences, Jan Dlugosz University, ul. Waszyngtona 4/8, 42-200 Częstochowa, Poland; 2 Medical Rehabilitation Center “Troniny”, ul. Stanislawa Staszica 34, 42-100 Klobuck, Poland; 3 Department of Rehabilitation, University of Medical Sciences in Poznan, Fredry 10, 61-701 Poznan, Poland

**Keywords:** Scoliosis, Adams test, Cobb angle, Angle of trunk inclination

## Abstract

**Background:**

Early detection of idiopathic scoliosis is one factor in determining treatment effectiveness. Therefore, the aim of this study was to assess the importance of the size of the trunk inclination angle (ATI) for the early detection of scoliosis in preschool- and school-age children, taking into account the location and size of the spine curvature.

**Methods:**

The study included a group of 216 children (mean age 11.54 years, standard deviation ± 3.05), who had previously untreated idiopathic scoliosis and a Cobb angle of ≥ 10°. The ATI values were compared with the corresponding Cobb angle values. The results of the ATI-Cobb correlation were compared to the ATI thresholds of 5° and 7°.

**Results:**

In the age groups 6–9, 10–12 and 13–17 years, the method sensitivity for the ATI ≥ 7° criterion was low at 33.90%, 27.69% and 51.29% (p < 0.05), respectively, while for the ATI ≥ 5° criterion, it was 67.8%, 69.23% and 93.48% (p < 0.05), respectively. With respect to location, significantly more frequent misdiagnoses (p < 0.05) were related to the lumbar and thoracolumbar (regions) sections of the spine in the groups aged 6–9 and 10–12 for ATI ≥ 7°; while no significant relationship was found at ATI ≥ 5°. For both ATI levels, the most frequent cases of mis- or undiagnosed scoliosis were observed among children with a Cobb angle of 10°-14° (*p* = 0.004).

**Conclusion:**

A low predictive ATI value was demonstrated regarding scoliosis detection for the ATI 7° criterion in children aged 6–9 and 10–12 years, particularly for the lumbar and thoracolumbar locations. Adoption of the threshold of ATI 5° in screening tests for children aged 6–12 years, as well as for lower locations of scoliosis, may be more effective in the early detection of scoliosis.

Trial registration.

This study was approved by the Jan Dlugosz University in Czestochowa Ethics Committee KE-U/7/2021, and conducted under the Declaration of Helsinki.

**Supplementary Information:**

The online version contains supplementary material available at 10.1186/s12891-021-04965-4.

## Background

Early diagnosis of idiopathic scoliosis (IS) is an important factor determining the effectiveness of treatment [1, 2]. The primary criterion of scoliosis detection in a clinical examination is an assessment of the angle of trunk inclination (ATI) using the Adams test [2–5]. School screening for spinal dysfunction by experienced staff is a reliable method of early detection of scoliosis; however, a complete diagnosis is made through orthopedic, pediatric or physiotherapeutic examinations [6–10]. Despite significant progress in diagnosis and treatment, it is assumed that in 0.1% of children with scoliosis, the Cobb angle reaches over 40° [1]. One of the primary reasons for this is that a small percentage of diagnosed scoliosis cases is made at the initial stage of the disease, which is estimated to be only 30% to 78% of total cases [2, 11–13]. The premier research societies focusing on the problems of idiopathic scoliosis, including SRS, SOSORT, and IRSSD, determined the parameters and methods of clinical assessment, where the criterion of screening scoliosis detection is ATI ≥ 7°, and ATI ≥ 5° in specialist units, or the sum of ATI values, known as the “Hump Sum 8°” [1, 7, 12, 14]. In addition, the person’s sex, family history, biological age, individual dynamics of growth, physique, and geographical region of residence should be considered [11, 15–18].

Furthermore, adoption of different ATI criteria for screening and medical assessments aims at preventing overdiagnosis and unnecessary X-ray tests [4, 19, 20]. Thus, a clinical diagnosis of scoliosis is based on the putative ATI-Cobb correlation, which, in certain circumstances, may be prone to error [4, 11]. According to the authors’ observations, the likelihood of misdiagnosed scoliosis when following the ATI criteria may be affected by a child’s age and the location of developing scoliosis; but to date, these relationships have not been confirmed in the literature. Therefore, this study aimed to assess the reliability of the angle of trunk inclination (ATI) measurements concerning early detection of scoliosis depending on the age, location, and size of the curvature in preschool- and school-age children.

## Methods

This study was approved by the Jan Dlugosz University in Czestochowa Ethical Committee KE-U/7/2021 and conducted under the Declaration of Helsinki. All the parents of the subjects were kept informed of the purpose and process of examination and subsequently gave their written consent before the study.

The design of the cohort study utilized data collected from the children’s medical records in the scoliosis treatment center. These patients were referred there by family physicians, pediatricians, orthopedists or physiotherapists due to suspected scoliosis. The preliminary condition for inclusion into the study was the availability of a child’s current spinal X-ray image during the first medical examination. These patients’ data were used to compare the values of clinical parameters, particularly the ATI value, with the severity of scoliosis seen in X-ray images of untreated children. The examinations were performed between 2011 and 2019 by a physician with 25 years of experience, with a specialization in physical medicine and rehabilitation, and with medical rehabilitation experience using a protocol of body posture assessment applied within the institution.

To verify the accuracy of the ATI and Cobb angle measurements, an additional study was conducted by two medical doctors. One was the same person who conducted the main examination; the other was a medical doctor with 15 years of experience in this field. The additional ATI and Cobb angle measurements were performed three times each day, in a group of 21 people meeting the criteria of the study (age, gender distribution, and ATI and X-ray range), all of which were similar to the main study. These people were neither treated with a corset nor had any physical therapy between studies. The inter-class correlation coefficient and the intra-observer ICC were calculated; the intra-observer ICC for the ATI measurement ranged from 0.92–0.94, and for the Cobb angle it ranged from 0.96–0.98. The inter-observer ICC for the ATI measurement was 0.92, and for the Cobb angle was 0.96.

Study participants.

Among 889 patients treated in the center, 216 children aged 6–17 years met the inclusion criteria; the mean age was 11.54 years (SD ± 3.05) and comparable to the median value. Characteristics of the study group are presented in Table 1. The participants were assigned to three age groups: 6–9, 10–12, and 13–17 years; the largest group consisted of children in the 13–17 category (42.6%, *N* = 92). Selection of the study participants was based on the following inclusion criteria: availability of the child’s current (obtained within the previous 3 months) X-ray image during the first clinical assessment, revealing signs of idiopathic scoliosis with the Cobb angle of ≥ 10°; Risser ≤ 3; and age of 6 to 17 years. The exclusion criteria were as follows: incomplete data in medical records; previous scoliosis treatment (e.g. a brace), which may infer with the assessment of scoliosis; congenital disorders, such as shortening of one limb exceeding 2 cm; genetic conditions; neurological diseases related to the locomotor system; cardiovascular diseases; previous injuries or surgeries; neuromuscular conditions; and intellectual disability.

**Table 1:** The main characteristics of the patients

Variable	Parameter	Results
Age	Total	216
	Mean (SD)	11.54 (3.05)
	6–9	27.3% (*N* = 59)
	10–12	30.1% (*N* = 65)
	13–17	42.6% (*N* = 92)
Gender	Girls	79.6% (*N* = 172)
	Boys	20.4% (*N* = 44)
ATI [°] (ranges)		
	Mean (SD)	6.82 (3.4)
	3–4	20.8% (*N* = 45)
	5–6	39.8% (*N* = 86)
	≥ 7	39.4% (*N* = 85)
Cobb [°] (ranges)	Mean (SD)	22.03 (11.18)
	10–14	25.9% (*N* = 56)
	15–20	30.1% (*N* = 65)
	≥ 21	44% (*N* = 95)

Measurements.

The ATI in the Adams test was assessed using a Bunnell scoliometer [3]. The severity of scoliosis was measured using the Cobb angle according to the SOSORT guidelines [21].

### Data analysis

The ATI-Cobb relationship was determined, using two ATI criteria for the diagnosis of scoliosis: 5° [19] and 7° [2, 21]. The study group was divided into three age groups (6–9, 10–12, and 13–17 years), according to assumptions dictated by clinical practice. The obtained results were compiled for three ranges of the Cobb angle: 10°-14°, 15°-21° and above 21°, relating to the therapeutic procedures [1, 2, 21].

Statistics.

Data are presented as arithmetic mean, standard deviation, median, minimum and maximum values, as well as percent values. Normality for specific variables was determined using the Shapiro–Wilk test. For comparisons of intergroup variables, the nonparametric Mann–Whitney U test was applied. The chi-squared and Fisher’s tests were used to investigate relationships between the categorical variables. To study relationships between the ATI and X-ray variables, Spearman’s correlation coefficient was applied. The assumed statistical significance level was *p* = 0.05. The Youden index was used to assess the sensitivity and specificity of the test for age groups. R statistical software was used for all calculations and graphs.

## Results

Characteristics of the 216 study participants are presented in Table 1. There were more girls than boys in the study, and the largest age category was represented by the participants aged 13 to 17. Considering the numbers of participants in the age groups for three ATI ranges, approximately 20% of children demonstrated an ATI of 3–4°. Regarding the three ranges of the Cobb angle, the largest group consisted of children with a Cobb angle value of ≥ 21° (Table 1).

For further analyses, the study group was divided into two categories: ATI ≥ 7° and ATI ≥ 5°. Regarding the ATI ≥ 7° criterion, misdiagnoses (undiagnosed scoliosis cases) were significantly more frequent in the age groups of 6–9 and 10–12 years (chi-squared test, *p* = 0.0076), while for the ATI ≥ 5°, a significantly higher frequency of proper diagnoses was observed in all age groups (chi-squared test, p < 0.001). Regarding the location of scoliosis, a significantly higher frequency of misdiagnoses was related to the lumbar and thoracolumbar regions (Fisher’s test, *p* = 0.0214), while for ATI ≥ 5°, the frequency did not significantly differ between the two locations (Table 2).

**Table 2:** Comparison of variables from the patients’ characteristics concerning the ATI 7° and ATI 5° groups

Variable	Parameter	ATI 7° criterion			ATI 5° criterion		
		**ATI ≥ 7° (*N* = 85)**	**ATI < 7° (*N* = 131)**	***p*-value**	**ATI ≥ 5° (*N* = 171)**	**ATI < 5° (*N* = 45)**	***p*-value**
**Age (ranges)**	6–9 (*N* = 59)	33.9% (*N* = 20)	66.1% (*N* = 39)	**0.0076**	67.8% (*N* = 40)	32.2% (*N* = 19)	** < 0.001**
	10–12 (*N* = 65)	27.7% (*N* = 18)	72.3% (*N* = 47)		69.2% (*N* = 45)	30.8% (*N* = 20)	
	13–17 (*N* = 92)	51.1% (*N* = 47)	48.9% (*N* = 45)		93.5% (*N* = 86)	6.5% (*N* = 6)	
**Gender**	Girls (*N* = 172)	43% (*N* = 74)	57% (*N* = 98)	**0.0443**	80.8% (*N* = 139)	19.2% (*N* = 33)	0.3317
	Boys (*N* = 44)	25% (*N* = 11)	75% (*N* = 33)		72.7% (*N* = 32)	27.3% (*N* = 12)	
	Median (IQR)	26 (20—32)	26 (21—31)		26 (21—32)	26 (20—30.25)	
**Location of scoliosis**	Th – thoracic (*N* = 86)	50% (*N* = 43)	50% (*N* = 43)	**0.0214**	80.2% (*N* = 69)	19.8% (*N* = 17)	0.9462
	Th-L – thoraco-lumbar (*N* = 75)	33.3% (*N* = 25)	66.7% (*N* = 50)		78.7% (*N* = 59)	21.3% (*N* = 16)	
	L – lumbar (*N* = 54)	29.6% (*N* = 16)	70.4% (*N* = 38)		77.8% (*N* = 42)	22.2% (*N* = 12)	
**Cobb (ranges)**	10–14 (*N* = 56)	14.3% (*N* = 8)	85.7% (*N* = 48)	** < 0.001**	64.3% (*N* = 36)	35.7% (*N* = 20)	**0.002**
	15–20 (*N* = 65)	30.8% (*N* = 20)	69.2% (*N* = 45)		78.5% (*N* = 51)	21.5% (*N* = 14)	
	≥ 21 (*N* = 95)	60% (*N* = 57)	40% (*N* = 38)		88.4% (*N* = 84)	11.6% (*N* = 11)	
	Median (IQR)	10 (5—10)	7 (5—10)		7 (5.5—10)	7 (5—10)	

Significantly more misdiagnoses were observed in both groups within the Cobb angle ranges of 10–14° and 15–20°; the increase in the Cobb angle value was associated with a reduced rate of undiagnosed scoliosis cases. For both ATI ≥ 5° and ATI ≥ 7° criteria, the largest number of properly-diagnosed scoliosis cases was observed among patients with the Cobb angle ≥ 21° (*N* = 84) (chi-squared test, *p* = 0.002) (Table 2).

We observed a statistically significant relationship between the classification of patient X-ray findings and the ATI ≥ 7° criterion in the age groups of 10–12 (Fisher’s test, p < 0.01) and 13–17 (Fisher’s test, p < 0.001). In both age groups, the highest rate of falsely diagnosed scoliosis cases was demonstrated in the group with the Cobb angle of 10°-14° (Table 3).

**Table 3:** X-ray comparisons of ATI 7° and ATI 5° classification for specific age groups

6–9 years old				
**ATI 7° criterion**		**ATI ≥ 7° (*N* = 20)**	**ATI < 7° (*N* = 39)**	***p*-value**
**Cobb (ranges)**	10–14 (*N* = 21)	23.8% (*N* = 5)	76.2% (*N* = 16)	0.2569
	15–20 (*N* = 22)	31.8% (*N* = 7)	68.2% (*N* = 15)	
	≥ 21 (*N* = 16)	50% (*N* = 8)	50% (*N* = 8)	
**ATI 5° criterion**		**ATI ≥ 5° (*N* = 40)**	**ATI < 5° (*N* = 19)**	***p*-value**
**Cobb (ranges)**	10–14 (*N* = 21)	57.1% (*N* = 12)	42.9% (*N* = 9)	0.472
	15–20 (*N* = 22)	72.7% (*N* = 16)	27.3% (*N* = 6)	
	≥ 21 (*N* = 16)	75% (*N* = 12)	25% (*N* = 4)	
**10–12 years old**				
**ATI 7° criterion**		**ATI ≥ 7° (*N* = 18)**	**ATI < 7° (*N* = 47)**	***p*-value**
**Cobb (ranges)**	10–14 (*N* = 20)	5% (*N* = 1)	95% (*N* = 19)	**0.0042**
	15–20 (*N* = 23)	26.1% (*N* = 6)	73.9% (*N* = 17)	
	≥ 21 (*N* = 22)	50% (*N* = 11)	50% (*N* = 11)	
**ATI 5° criterion**		**ATI ≥ 5° (*N* = 45)**	**ATI < 5° (*N* = 20)**	***p*-value**
**Cobb (ranges)**	10–14 (*N* = 20)	55% (*N* = 11)	45% (*N* = 9)	0.277
	15–20 (*N* = 23)	73.9% (*N* = 17)	26.1% (*N* = 6)	
	≥ 21 (*N* = 22)	77.3% (*N* = 17)	22.7% (*N* = 5)	
**13–17 years old**				
**ATI 7° criterion**		**ATI ≥ 7° (*N* = 47)**	**ATI < 7° (*N* = 45)**	***p*-value**
**Cobb (ranges)**	10–14 (*N* = 15)	13.3% (*N* = 2)	86.7% (*N* = 13)	** < 0.001**
	15–20 (*N* = 20)	35% (*N* = 7)	65% (*N* = 13)	
	≥ 21 (*N* = 57)	66.7% (*N* = 38)	33.3% (*N* = 19)	
**ATI 5° criterion**		**ATI ≥ 5° (*N* = 86)**	**ATI < 5° (*N* = 6)**	***p*-value**
**Cobb (ranges)**	10–14 (*N* = 15)	86.7% (*N* = 13)	13.3% (*N* = 2)	0.1641
	15–20 (*N* = 20)	90% (*N* = 18)	10% (*N* = 2)	
	≥ 21 (*N* = 57)	96.5% (*N* = 55)	3.5% (*N* = 2)	

Concerning the location of scoliosis and the ATI ≥ 7° criterion, there was a statistically significant relationship between the classification of patient X-ray findings for all spineal regions (Fisher’s test, p < 0.01). In the case of scoliosis affecting the thoracic and thoracolumbar region, the rate of patients with falsely diagnosed scoliosis decreased with increasing Cobb angle values. Regarding the lumbar region, the highest rate of falsely diagnosed cases was related to the children with Cobb angle values between 15° and 20°. For the ATI ≥ 5° criterion, proper diagnoses were significantly more frequent, and there were no cases of ATI < 5° among the patients with the Cobb angle ≥ 21° (Table 4).

**Table 4:** X-ray comparisons regarding ATI 7° and ATI 5° classification for the specific locations

Th – thoracic region				
**ATI 7° criterion**		**ATI ≥ 7° (*N* = 43)**	**ATI < 7° (*N* = 43)**	***p*-value**
**Cobb (ranges)**	10–14 (*N* = 17)	17.6% (*N* = 3)	82.4% (*N* = 14)	0.004
	15–20 (*N* = 24)	45.8% (*N* = 11)	54.2% (*N* = 13)	
	≥ 21 (*N* = 45)	64.4% (*N* = 29)	35.6% (*N* = 16)	
**ATI 5° criterion**		**ATI ≥ 5° (*N* = 69)**	**ATI < 5° (*N* = 17)**	***p*-value**
**Cobb (ranges)**	10–14 (*N* = 17)	58.8% (*N* = 10)	41.2% (*N* = 7)	0.0708
	15–20 (*N* = 24)	87.5% (*N* = 21)	12.5% (*N* = 3)	
	≥ 21 (*N* = 45)	84.4% (*N* = 38)	15.6% (*N* = 7)	
**Th-L – thoracolumbar region**				
**ATI 7° criterion**		**ATI ≥ 7° (*N* = 25)**	**ATI < 7° (*N* = 50)**	***p*-value**
**Cobb (ranges)**	10–14 (*N* = 27)	14.8% (*N* = 4)	85.2% (*N* = 23)	0.004
	15–20 (*N* = 26)	30.8% (*N* = 8)	69.2% (*N* = 18)	
	≥ 21 (*N* = 22)	59.1% (*N* = 13)	40.9% (*N* = 9)	
**ATI 5° criterion**		**ATI ≥ 5° (*N* = 59)**	**ATI < 5° (*N* = 16)**	***p*-value**
**Cobb (ranges)**	10–14 (*N* = 27)	66.7% (*N* = 18)	33.3% (*N* = 9)	0.004
	15–20 (*N* = 26)	73.1% (*N* = 19)	26.9% (*N* = 7)	
	≥ 21 (*N* = 22)	100% (*N* = 22)	0% (*N* = 0)	
**L – lumbar**				
**ATI 7° criterion**		**ATI ≥ 7° (*N* = 16)**	**ATI < 7° (*N* = 38)**	***p*-value**
**Cobb (ranges)**	10–14 (*N* = 12)	8.3% (*N* = 1)	91.7% (*N* = 11)	0.0015
	15–20 (*N* = 15)	6.7% (*N* = 1)	93.3% (*N* = 14)	
	≥ 21 (*N* = 27)	51.9% (*N* = 14)	48.1% (*N* = 13)	
**ATI 5° criterion**		**ATI ≥ 5° (*N* = 42)**	**ATI < 5° (*N* = 12)**	***p*-value**
**Cobb (ranges)**	10–14 (*N* = 12)	66.7% (*N* = 8)	33.3% (*N* = 4)	0.3605
	15–20 (*N* = 15)	73.3% (*N* = 11)	26.7% (*N* = 4)	
	≥ 21 (*N* = 27)	85.2% (*N* = 23)	14.8% (*N* = 4)	

The sensitivity of the method for the ATI 7° criterion was the lowest in the groups of children aged 6–12, while for the ATI ≥ 5° criterion, sensitivity was at an acceptable or higher level. Considering both ATI criteria, the method is definitively more effective among older children (Table 5).

**Table 5:** Diagnostic method assessment based on ATI measurement criteria concerning detection of scoliosis regarding age. CI stands for a confidence interval

	**ATI 7° criterion**				**ATI 5° criterion**			
	**Sensitivity [%]**	**Specificity [%]**	**PPV [%]**	**NPV [%]**	**Sensitivity [%]**	**Specificity [%]**	**PPV [%]**	**NPV [%]**
**Overall**								
Value	39.35	84.61	97.70	7.74	79.17	30.77	95.00	8.16
95% CI	32.79–46.20	54.55—98.07	91.94—99.72	3.93—13.43	73.13–84.38	9.09–61.43	90.72–97.67	2.27–19.60
**6 to 9 years old**								
Value	33.90	80.00	95.24	9.30	67.8	20.00	90.90	5.00
95% CI	22.09–47.39	28.36–99.49	76.18–99.98	2.59–22.14	54.36– 79.38	50.51–71.64	78.33–97.47	0.13–24.87
**10 to 12 years old**								
Value	27.69	100.00	100.00	9.61	69.23	60.00	95.74	13.04
95% CI	17.31–40.19	47.82–100.00	81.47–100.00	3.20–21.03	56.55–80.09	14.66–94.73	85.46–99.48	2.78–33.59
**13 to 17 years old**								
Value	51.29	66.67	97.92	4.26	93.48	0.0	96.63	0.0
95% CI	40.44–61.66	9.43–99.16	88.93–99.95	0.05–14.54	86.34–97.57	0.0–70.76	90.46–99.30	0.0–45.93

The lowest level of sensitivity for the ATI ≥ 7° criterion was associated with the lumbar and thoracic-lumbar locations, while for the ATI ≥ 5° criterion, sensitivity was over two times greater in these regions (Table 6).

**Table 6:** Diagnostic method assessment based on ATI measurements concerning detection of scoliosis regarding location. CI stands for a confidence interval

	**ATI 7° criterion**				**ATI 5° criterion**			
	**Sensitivity [%]**	**Specificity [%]**	**PPV [%]**	**NPV [%]**	**Sensitivity [%]**	**Specificity [%]**	**PPV [%]**	**NPV [%]**
**Th – thoracic region**								
Value	50.00	100.00	100.0	6.52	80.23	66.67	98.57	10.52
95% CI	39.18–60.98	29.24–100.00	91.78–100.0	1.37–17.90	70.25–88.04	9.43–99.16	92.30–99.96	1.30–33.14
**Th-L – thoracolumbar region**								
Value	33.33	77.78	92.59	12.28	78.67	11.11	88.06	5.88
95% CI	22.86–45.17	39.88–97.19	75.71–99.09	5.08–23.67	67.68–87.29	0.28–48.25	77.82–94.70	0.15–28.69
**L – lumbar region**								
Value	29.62	100.00	100.00	2.56	77.78	100.00	100.00	7.69
95% CI	17.98–43.61	2.50–100.00	79.41–100.00	0.06–13.48	64.40–87.96	2.5–100.00	91.59–100.00	0.19–36.03

As a result of the study, an average positive relationship between the measured values of the ATI and Cobb angles was observed. These findings refer to the whole study group, the specific age groups, and the trunk inclination locations of interest.

The sensitivity and specificity of the method, estimated using the Youden index, confirmed low sensitivity in the age range of 6–9 years, and in the lumbar and thoracic-lumbar locations. Despite high sensitivity, low index values recorded for the ATI ≥ 5° threshold resulted from the low specificity of the test (Table 7).

**Table 7:** The assessment of sensitivity and specificity of the Adams test, according to the Youden index

Category	Positive	False positive	False negative	Negative	Sensitivity	Specificity	Youden index
**Distribution criterion ATI ≥ 7°**							
Overall	85	2	131	11	39.4%	84.6%	24.0%
6–9	20	1	39	4	33.9%	80.0%	13.9%
10–12	18	0	47	5	27.7%	100%	27.7%
13–17	47	1	45	2	51.1%	66.67%	17.8%
**Distribution criterion ATI ≥ 5°**							
Overall	171	9	45	4	79.2%	30.77%	9.9%
6–9	40	4	19	1	67.8%	20%	12.2%
10–12	45	2	20	3	69.2%	60%	29.2%
13–17	86	3	6	0	93.5%	0%	6.5%

**Fig. 1:**
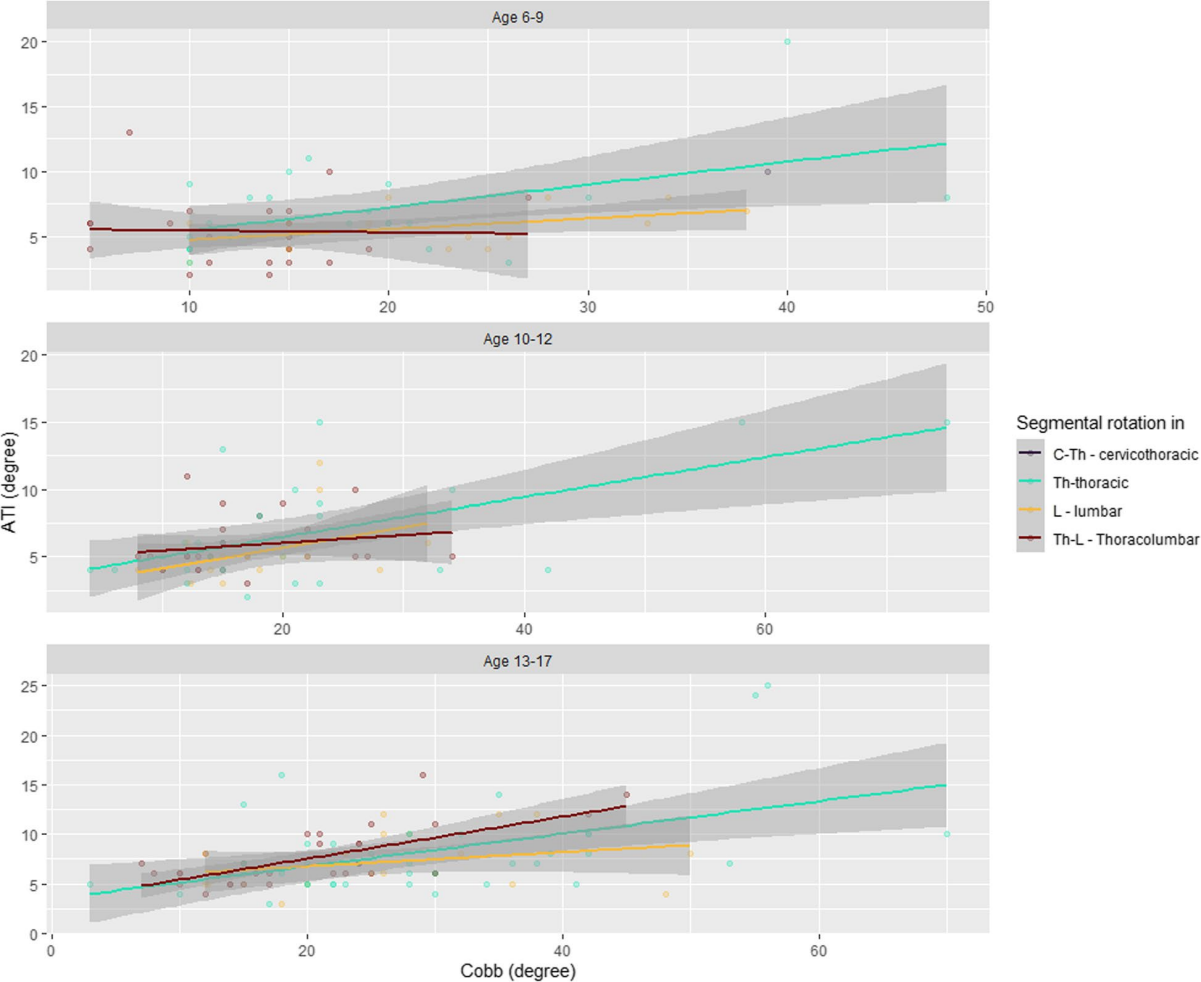
Correlation of the ATI and the Cobb angles, depending on the location of scoliosis

The correlation between the ATI and Cobb angle for different age groups and different curvature positions is shown in Fig. 1.

## Discussion

A clinical method based on the ATI measurement using the Adams test estimates the correlation between concomitant spinal deformities in the frontal and horizontal planes, and their severity strictly determines the predictive ATI value in scoliosis detection [1, 2, 5, 6]. Many publications confirm a high ATI/Cobb correlation [2, 3, 5, 13]; however, recent reports suggest that patient age and location of scoliosis should be considered to avoid diagnostic error in screening tests, based on the assumed screening ATI criteria [11, 22, 23]. In the presented material, significant correlation differences are demonstrated, dependent upon a child’s age.

In the study of children aged 6–9 and 10–12 years, with scoliosis detected using X-ray, those with ATI < 7° constituted the majority of cases (66% for ages 6–9, and 72% for ages 10–12). However, using the 5° threshold, the percentages are only 32% and 28%, respectively, with this being a statistically significant difference (Table 2).

The analysis of the Cobb angle in the test group using the 7° threshold shows that scoliosis is most difficult to detect when the Cobb angle is between 10°-14° and 15°-20°, situations that occur in the early stages of scoliosis (Table 3). Scoliosis is easiest to detect using the Adams test in children aged 13–17 years, with a Cobb angle of ≥ 21°; for these children, the percentage of correct diagnoses ranged from 79.2% to 93.5%.

The low percentage of correct diagnoses in the Adams test, in relation to the child’s age and the size of the Cobb angle for the ATI threshold ≥ 7°, may suggest its low predictive value for early diagnosis of scoliosis in young children. Earlier studies confirmed weaker ATI-Cobb correlations in children aged 6–9 years, especially in the lumbar region [7, 11, 23–25].

In the present study, analysis of the impact of scoliosis location on the predictive value of the Adams test showed that children with curvatures in the lumbar and thoracolumbar spine had the lowest chance of detecting scoliosis using the ATI threshold of ≥ 7°. Among younger children, in which the Cobb angle was 10°-14°, only 33% of children had a chance of being diagnosed with scoliosis. Older children (13–16 years) were slightly more likely to be diagnosed (50%), especially those with a Cobb angle of ≥ 21° (64%). Thus, the older a child’s age and the greater the Cobb angle, the higher the predictive value of the test (Table 3).

Earlier studies describing the ATI-Cobb correlation depending on the location of spinal curvature confirm the weakest correlation in the lumbar region [25, 26]. Moreover, reports from the analysis of the correlation of vertebral rotation and Cobb angle in X-ray and CT imaging also confirm a low correlation in the lumbar region (coefficient’s range of 0.48–0.70) [27]. The same author assessed the compliance of ATI with radiological measurements of epiphyseal rotation, and found the lowest correlation in the lumbar region (coefficient’s range of 0.32–0.46) [27]. In some studies, the Cobb angle of 25° corresponded to ATIs of 7° and 6° in the thoracic-lumbar and lumbar sections of the spine, respectively, with the ATI-Cobb correlation coefficient range of 0.57–0.65 [28].

Among the primary reasons for a low ATI-Cobb correlation and, consequently, the high percentage of late scoliosis diagnoses in children, were the examiner’s diligence and skills [29] and the examiner’s experience [13].

In this study, to evaluate measurement accuracy, the interobserver correlation coefficient was determined, which for ATI measurements ranged from 0.92–0.94, and for the Cobb angle ranged from 0.96–0.98. The intra-observer correlation coefficient for the ATI measurement was 0.92, and for the Cobb angle it was 0.96. Such high intra- and inter-observer coefficients in comparison to other reports are related to the fact that the study was conducted by two researchers with many years of experience (25 and 15 years, respectively).

Moreover, it is worth noting that in publications that utilize with intra-class correlation coefficients (ICCs) in screening tests, the intra- and inter-observer ICCs were 0.61 and 0.29 for the thoracic and lumbar spine, respectively [11].

It follows that in the assessment of the lumbar section, errors related to low ATI-Cobb correlation, as well as the ATI measurement error itself, may add up [30, 31].

In the present results, undiagnosed scoliosis was most frequent in the group of younger children (6–12 years) with a Cobb angle of 10°-14°, a situation in which the sensitivity of the test was the lowest (27.69–33.90). In the study with the ATI threshold of ≥ 5°, the sensitivity of the Adams test was high for both the thoracic (80.2%) and the lumbar (77.8%) sections.

However, the specificity of the test was low, as illustrated by the Youden index, which for the 5° threshold was still low. This suggests that ATI should not be used as the only criterion to diagnose scoliosis and qualify patients for treatment (Table 7). This means that to increase the percentage of early-stage scoliosis diagnoses, the ATI threshold should be reduced to 5° for the abovementioned age group in the lumbar and thoracolumbar locations, while for the remaining locations and age ranges, the ATI threshold of 7° should be maintained.

When an X-ray image of the spine is available, we can use a well-developed prognostic model, taking into account Cobb’s angle, age, sex, etc. [31], while the correct interpretation of the clinical examination in the doctor’s office appears to be the most important. The study opens up a discussion as to whether the adoption of two ATI reference values ​​should also apply to the screening process.

Difficulties in the clinical diagnosis of scoliosis result from morphological differences in the three-dimensional spinal deformity. These differences are specific to each curvature, and are associated with nonspecific symptoms [32–38].

One of the limitations of this study is the fact that our cohort came from only one site. To solve this problem, a multicentric study is planned.

## Conclusions

A low predictive value of the trunk inclination angle measurements was demonstrated regarding scoliosis detection for the ATI ≥ 7° criterion in children aged 6–9 and 10–12, particularly for the lumbar and thoracolumbar locations. Adoption of the ATI ≥ 5° threshold in screening tests for children aged 6–12, and for lower spinal locations, may be more effective in early detection of this scoliosis.

## Supplementary Information

Below is the link to the electronic supplementary material.

Additional file1 (XLSX 267 kb)

## Data Availability

All data generated or analysed during this study are included in this published article and its supplementary information files.

## Abbreviations

**ATI**: Angle of trunk inclination
**IS**: Idiopathic scoliosis
**SRS**: Scoliosis Research Society
**SOSORT**: Scoliosis Orthopedic and Rehabilitation Treatment
**IRSSD**: International Research Society of Spinal Deformities
